# Sex disparities in adverse outcomes after surgically managed isolated traumatic spinal injury

**DOI:** 10.1007/s00068-023-02275-z

**Published:** 2023-05-16

**Authors:** Ahmad Mohammad Ismail, Maximilian Peter Forssten, Babak Sarani, Marcelo A. F. Ribeiro, Parker Chang, Yang Cao, Frank Hildebrand, Shahin Mohseni

**Affiliations:** 1 Department of Orthopedic Surgery, Orebro University Hospital, 701 85 Orebro, Sweden; 2 School of Medical Sciences, Orebro University, 702 81 Orebro, Sweden; 3 Surgery and Emergency Medicine, Center of Trauma and Critical Care, George Washington University, Washington, DC USA; 4 Surgery, Pontifical Catholic University of São Paulo, São Paulo, Brazil; 5 Surgery, Khalifa University and Gulf Medical University, Abu Dhabi, United Arab Emirates; 6 Division of Trauma, Critical Care & Acute Care Surgery, Department of Surgery, Sheikh Shakhbout Medical City, Mayo Clinic, Abu Dhabi, United Arab Emirates; 7 Center for Trauma and Critical Care, Department of Surgery, George Washington University, Washington, DC USA; 8 Clinical Epidemiology and Biostatistics, Faculty of Medicine and Health, School of Medical Sciences, Orebro University, 701 82 Orebro, Sweden; 9 Department of Orthopedics, Trauma and Reconstructive Surgery, University Hospital RWTH Aachen, Pauwelsstrasse 30, 52074 Aachen, Germany

**Keywords:** Spinal injury, Sex disparity, Equity, Mortality, Morbidity

## Abstract

**Background:**

Traumatic spinal injury (TSI) encompasses a wide range of injuries affecting the spinal cord, nerve roots, bones, and soft tissues that result in pain, impaired mobility, paralysis, and death. There is some evidence suggesting that women may have different physiological responses to traumatic injury compared to men; therefore, this study aimed to investigate if there are any associations between sex and adverse outcomes following surgically managed isolated TSI.

**Methods:**

Using the 2013–2019 TQIP database, all adult patients with isolated TSI, defined as a spine AIS ≥ 2 with an AIS ≤ 1 in all other body regions, resulting from blunt force trauma requiring spinal surgery, were eligible for inclusion in the study. The association between the sex and in-hospital mortality as well as cardiopulmonary and venothromboembolic complications was determined by calculating the risk ratio (RR) after adjusting for potential confounding using inverse probability weighting.

**Results:**

A total of 43,756 patients were included. After adjusting for potential confounders, female sex was associated with a 37% lower risk of in-hospital mortality [adjusted RR (95% CI): 0.63 (0.57–0.69), p < 0.001], a 27% lower risk of myocardial infarction [adjusted RR (95% CI): 0.73 (0.56–0.95), p = 0.021], a 37% lower risk of cardiac arrest [adjusted RR (95% CI): 0.63 (0.55–0.72), p < 0.001], a 34% lower risk of deep vein thrombosis [adjusted RR (95% CI): 0.66 (0.59–0.74), p < 0.001], a 45% lower risk of pulmonary embolism [adjusted RR (95% CI): 0.55 (0.46–0.65), p < 0.001], a 36% lower risk of acute respiratory distress syndrome [adjusted RR (95% CI): 0.64 (0.54–0.76), p < 0.001], a 34% lower risk of pneumonia [adjusted RR (95% CI): 0.66 (0.60–0.72), p < 0.001], and a 22% lower risk of surgical site infection [adjusted RR (95% CI): 0.78 (0.62–0.98), p < 0.032], compared to male sex.

**Conclusion:**

Female sex is associated with a significantly decreased risk of in-hospital mortality as well as cardiopulmonary and venothromboembolic complications following surgical management of traumatic spinal injuries. Further studies are needed to elucidate the cause of these differences.

**Supplementary Information:**

The online version contains supplementary material available at 10.1007/s00068-023-02275-z.

## Introduction

Traumatic spinal injury (TSI) encompasses a broad spectrum of injuries that affect the spinal cord, nerve roots, osseous structures, and soft tissues of the spinal column. These injuries may result from a wide range of high-energy and low-energy mechanisms. The clinical manifestations of TSI include pain, impaired mobility, and mechanical instability of the spinal column, as well as partial or complete paralysis. In severe cases, TSI or related complications may result in death [1]. Previous research has suggested that females may have different physiological responses to traumatic injury compared to their male counterparts, which may impact outcomes pursuant to injury [2–10]. However, the majority of studies conducted on this subject have made use of animal models, and when attempting to translate these associations to human patients with traumatic injuries, the results have been inconsistent. The primary focus of research on sex disparities in spinal injuries has largely been on differences in functional outcomes and mortality in patients with spinal *cord* injury (SCI) [11–18]. However, not all TSI patients suffer a SCI. Therefore, this study aimed to investigate if there are any associations between biological sex and adverse outcomes following surgically managed isolated TSI.

## Materials and methods

### Data source and study population

Data for the current study was retrieved from The American College of Surgeons Trauma Quality Improvement Program (TQIP) database. Data retrieved included: age, sex, race, the abbreviated injury severity score (AIS) for each body region, comorbidities, presence of an advanced directive limiting care, injury characteristics, type of surgery, discharge disposition, and complications. All adult patients (18 years or older) registered in TQIP between 2013 and 2019 who suffered an isolated spine injury as a result of a blunt trauma and were managed surgically were included. An isolated spine injury was defined as any spine AIS ≥ 2 and an AIS ≤ 1 in the remaining regions. Patients were excluded if their sex was not recorded in the dataset or if they had a spine AIS of 6, as these injuries are not generally considered survivable. The need for ethical approval by an institutional review board was waived for the current investigation as all analyses were performed using an anonymized, retrospective dataset. The Strengthening the Reporting of Observational Studies in Epidemiology (STROBE) guidelines and Declaration of Helsinki were adhered to throughout the completion of the investigation [19].

### Statistical analysis

Owing to continuous variables being non-normally distributed, they were summarized as medians and interquartile ranges (IQRs), with the Mann–Whitney U-test being used to determine the statistical significance of differences between groups. Categorical variables were presented as counts and percentages. Either the Chi-squared test or Fisher’s exact test was used, as appropriate, to determine the statistical significance of differences in the distribution between groups. The primary outcome of interest was in-hospital mortality, with the secondary outcome consisting of in-hospital complications (myocardial infarction, cardiac arrest with cardiopulmonary resuscitation (CPR), stroke, deep vein thrombosis, pulmonary embolism, acute respiratory distress syndrome (ARDS), pneumonia, and surgical site infection).

To adjust for potential confounding, inverse probability weighting (IPW) was used to balance the confounders between males and females. The probability was determined using a logistic regression model; sex was set as the response variable while the explanatory variables consisted of age, race, AIS score in each region, level of injury, presence of SCI, level of spine surgery, comorbidities (hypertension, previous myocardial infarction, congestive heart failure, history of peripheral vascular disease, cerebrovascular disease, dementia, chronic obstructive pulmonary disease, smoking status, chronic renal failure, diabetes mellitus, cirrhosis, coagulopathy, currently receiving chemotherapy for cancer, metastatic cancer, drug use disorder, alcohol use disorder, major psychiatric illness), and advanced directives limiting care. The weights were calculated as $\frac{1}{probability \; of \; female \; sex}$ for female patients and $\frac{1}{1 - probability \; of \; female \; sex}$ for male patients. After IPW, differences in demographics and clinical characteristics between males and females were assessed by employing absolute standardized differences (ASDs), where an ASD < 0.1 was considered balanced. The risk ratio (RR) of the outcomes and corresponding confidence interval (CI) were then determined based on the weighted counts [20].

Statistical significance was defined as a two-sided p-value less than 0.05. Among patients included in the current dataset, < 2% had any form of missing data. Subsequently, data that were missing were assumed to be missing at random. To manage missing data, multiple imputation by chained equations was employed; logistic regression was used for race and a proportional odds model for the spine AIS. Analyses were performed using the tidyverse, haven, mice, survey, tableone, and writexl packages in the statistical software R 4.0.5 (R Foundation for Statistical Computing, Vienna, Austria) [21].

## Results

After applying the inclusion and exclusion criteria, 43,756 patients remained for further analysis; 68% were male (N = 29,739) and 32% were female (N = 14,017). On average, women were older (59 vs 55 years, p < 0.001) and more often White (80.8% vs 76.0%, p < 0.001). Female patients were also more likely to suffer from hypertension (40.7% vs 37.1%, p < 0.001), dementia (3.6% vs 2.1%, p < 0.001), chronic obstructive pulmonary disease (7.7% vs 6.0%, p < 0.001), and major psychiatric illnesses (15.9% vs 8.3%, p < 0.001). Conversely, female patients were less likely to have previously suffered a myocardial infarction (0.7% vs 1.1%, p < 0.001) or suffer from cirrhosis (0.7% vs 0.9%, p = 0.023), drug use disorder (4.7% vs 7.2%, p < 0.001), or alcohol use disorder (5.2% vs 10.1%, p < 0.001) (Table 1). Female patients also tended to be less severely injured than male patients (Spine AIS 4 and 5: 19.2% vs 28.5%, p < 0.001). Mirroring this, cervical spine injuries were less prevalent among females (55.6% vs 63.9%, p < 0.001); consequently, cervical spinal cord injuries were also less common (24.6% vs 34.9%, p < 0.001), along with cervical spine surgery (69.6% vs 74.5%, p < 0.001), in female patients compared to male patients (Table 2).

**Table 1:** Demographics of patients with isolated traumatic spine injuries requiring surgery requiring surgery

	Male (N = 29,739)	Female (N = 14,017)	P-value
Age, median [IQR]	55 [38–68]	59 [41–73]	< 0.001
Race, n (%)			
White	22,607 (76.0)	11,320 (80.8)	< 0.001
Black	3816 (12.8)	1263 (9.0)	< 0.001
Asian	549 (1.8)	400 (2.9)	< 0.001
American Indian	245 (0.8)	120 (0.9)	0.773
Pacific islander	90 (0.3)	31 (0.2)	0.156
Other	1930 (6.5)	671 (4.8)	< 0.001
Missing	310 (1.0)	143 (1.0)	
Hypertension, n (%)	11,044 (37.1)	5704 (40.7)	< 0.001
Previous myocardial infarction, n (%)	314 (1.1)	94 (0.7)	< 0.001
Congestive heart failure, n (%)	1008 (3.4)	519 (3.7)	0.102
History of peripheral vascular disease, n (%)	206 (0.7)	91 (0.6)	0.649
Cerebrovascular disease, n (%)	591 (2.0)	303 (2.2)	0.243
Dementia, n (%)	613 (2.1)	503 (3.6)	< 0.001
COPD, n (%)	1788 (6.0)	1079 (7.7)	< 0.001
Current smoker, n (%)	7602 (25.6)	2755 (19.7)	< 0.001
Chronic renal failure, n (%)	416 (1.4)	167 (1.2)	0.085
Diabetes mellitus, n (%)	5123 (17.2)	2405 (17.2)	0.869
Cirrhosis, n (%)	278 (0.9)	100 (0.7)	0.023
Coagulopathy, n (%)	986 (3.3)	467 (3.3)	0.953
Currently receiving chemotherapy for cancer, n (%)	84 (0.3)	41 (0.3)	0.930
Metastatic cancer, n (%)	172 (0.6)	82 (0.6)	0.986
Drug use disorder, n (%)	2132 (7.2)	660 (4.7)	< 0.001
Alcohol use disorder, n (%)	3005 (10.1)	732 (5.2)	< 0.001
Major psychiatric illness, n (%)	2463 (8.3)	2230 (15.9)	< 0.001
Advanced directive limiting care, n (%)	584 (2.0)	405 (2.9)	< 0.001

*COPD* chronic obstructive pulmonary disease

**Table 2:** Clinical characteristics of patients with isolated traumatic spine injuries requiring surgery

	Male (N = 29,739)	Female (N = 14,017)	P-value
ISS, median [IQR]	9.0 [5.0–16]	9.0 [5.0–13]	< 0.001
Missing, n (%)	11 (0.0)	5 (0.0)	
Head AIS, n (%)			< 0.001
Injury not present	25,283 (85.0)	12,353 (88.1)	
1	4456 (15.0)	1664 (11.9)	
Face AIS, n (%)			< 0.001
Injury not present	24,363 (81.9)	11,754 (83.9)	
1	5376 (18.1)	2263 (16.1)	
Neck AIS, n (%)			0.020
Injury not present	29,364 (98.7)	13,801 (98.5)	
1	375 (1.3)	216 (1.5)	
Spine AIS, n (%)			< 0.001
2	10,923 (36.7)	5371 (38.3)	
3	10,309 (34.7)	5945 (42.4)	
4	6133 (20.6)	2080 (14.8)	
5	2359 (7.9)	616 (4.4)	
Missing	15 (0.1)	5 (0.0)	
Thorax AIS, n (%)			0.010
Injury not present	27,878 (93.7)	13,229 (94.4)	
1	1861 (6.3)	788 (5.6)	
Abdomen AIS, n (%)			0.097
Injury not present	28,937 (97.3)	13,599 (97.0)	
1	802 (2.7)	418 (3.0)	
Upper extremity AIS, n (%)			< 0.001
Injury not present	26,453 (89.0)	12,656 (90.3)	
1	3286 (11.0)	1361 (9.7)	
Lower extremity AIS, n (%)			0.330
Injury not present	26,917 (90.5)	12,645 (90.2)	
1	2822 (9.5)	1372 (9.8)	
External/Other AIS, n (%)			0.014
Injury not present	28,435 (95.6)	13,474 (96.1)	
1	1304 (4.4)	543 (3.9)	
Level of spine injury, n (%)			
Cervical	19,018 (63.9)	7796 (55.6)	< 0.001
Thoracic	7943 (26.7)	4115 (29.4)	< 0.001
Lumbar	7684 (25.8)	4225 (30.1)	< 0.001
Spinal cord injury, n (%)			
Cervical	10,378 (34.9)	3453 (24.6)	< 0.001
Thoracic	1722 (5.8)	717 (5.1)	0.004
Lumbar	1385 (4.7)	620 (4.4)	0.286
Level of spine surgery, n (%)			
Cervical	22,150 (74.5)	9752 (69.6)	< 0.001
Thoracic	15,842 (53.3)	8013 (57.2)	< 0.001
Lumbar	13,159 (44.2)	6787 (48.4)	< 0.001

*ISS* injury severity score, *AIS* abbreviated injury severity score

Prior to adjustment, female patients exhibited lower rates of in-hospital mortality (1.5% vs 2.5%, p < 0.001) as well as all studied complications, except for myocardial infarction, stroke, and surgical site infections, compared to males (Table 3). All covariates were balanced with an ASD < 0.1 after IPW (Supplemental Table 1). After adjustment for potential confounding, female sex was associated with a 37% lower risk of in-hospital mortality [adjusted RR (95% CI): 0.63 (0.57–0.69), p < 0.001], a 27% lower risk of myocardial infarction [adjusted RR (95% CI): 0.73 (0.56–0.95), p = 0.021], a 37% lower risk of cardiac arrest [adjusted RR (95% CI): 0.63 (0.55–0.72), p < 0.001], a 34% lower risk of deep vein thrombosis [adjusted RR (95% CI): 0.66 (0.59–0.74), p < 0.001], a 45% lower risk of pulmonary embolism [adjusted RR (95% CI): 0.55 (0.46–0.65), p < 0.001], a 36% lower risk of ARDS [adjusted RR (95% CI): 0.64 (0.54–0.76), p < 0.001], a 34% lower risk of pneumonia [adjusted RR (95% CI): 0.66 (0.60–0.72), p < 0.001], and a 22% lower risk of surgical site infection [adjusted RR (95% CI): 0.78 (0.62–0.98), p = 0.032], compared to their male counterparts. There was no statistically significant association between biological sex and risk of stroke after TSI (Table 4).

**Table 3:** Crude outcomes in patients with isolated traumatic spine injuries requiring surgery

	Male (N = 29,739)	Female (N = 14,017)	P-value
In-hospital mortality, n (%)	752 (2.5)	214 (1.5)	< 0.001
Myocardial infarction, n (%)	82 (0.3)	30 (0.2)	0.275
Cardiac arrest with CPR, n (%)	391 (1.3)	96 (0.7)	< 0.001
Stroke, n (%)	69 (0.2)	30 (0.2)	0.793
Deep vein thrombosis, n (%)	540 (1.8)	142 (1.0)	< 0.001
Pulmonary embolism, n (%)	259 (0.9)	63 (0.4)	< 0.001
Acute respiratory distress syndrome, n (%)	238 (0.8)	65 (0.5)	< 0.001
Pneumonia, n (%)	806 (2.7)	181 (1.3)	< 0.001
Surgical site infection, n (%)	115 (0.4)	38 (0.3)	0.068
Duration of ICU stay, median [IQR]	4.0 [3.0–8.0]	4.0 [3.0–6.0]	< 0.001

Duration of ICU stay is measured in days

*ICU* intensive care unit

**Table 4:** Risk ratio of mortality and complications after surgery in patients with isolated traumatic spine injuries, after IPTW

	Male	Female RR (95% CI)	P-value
In-hospital mortality	Reference	0.63 (0.57–0.69)	< 0.001
Myocardial infarction	Reference	0.73 (0.56–0.95)	0.021
Cardiac arrest with CPR	Reference	0.63 (0.55–0.72)	< 0.001
Stroke	Reference	0.95 (0.73–1.25)	0.749
Deep vein thrombosis	Reference	0.66 (0.59–0.74)	< 0.001
Pulmonary embolism	Reference	0.55 (0.46–0.65)	< 0.001
Acute respiratory distress syndrome	Reference	0.64 (0.54–0.76)	< 0.001
Pneumonia	Reference	0.66 (0.60–0.72)	< 0.001
Surgical site infection	Reference	0.78 (0.62–0.98)	0.032

All variables were balanced after IPW, with ASDs < 0.1. Weights are based on age, sex, race, highest abbreviated injury severity score in each region, level of injury, presence of spinal cord injury, level of spine surgery, hypertension, previous myocardial infarction, congestive heart failure, history of peripheral vascular disease, cerebrovascular disease, dementia, chronic obstructive pulmonary disease, smoking status, chronic renal failure, diabetes mellitus, cirrhosis, coagulopathy, currently receiving chemotherapy for cancer, metastatic cancer, drug use disorder, alcohol use disorder, major psychiatric illness, and advanced directives limiting care

*IPW* Inverse probability weighting, *RR* Risk ratio, *CI* Confidence Interval, *ASD* Absolute standardized difference

## Discussion

In this study, utilizing a large national trauma database including more than 40,000 surgically managed isolated TSI cases, it was found that female sex was associated with significantly lower risk of in-hospital morbidity and mortality. These associations were present both before and adjusting for confounding. This study highlights the need for further investigations into possible mechanisms explaining these findings.

Several previous publications suggest that females demonstrate better outcomes following severe trauma when compared to males [2, 9, 10, 22]. A notable consequence of traumatic injury is a disruption in the immune system, characterized by an aberration in the production of cytokines, chemokines, and other inflammatory mediators [2, 22]. It has been proposed that variation in hormone levels between males and females may account for discrepancies in physiological and immunological responses, with beneficial effects of estrogen and unfavorable consequences of (dihydro-) testosterone. These might ultimately affect the outcomes of the patients [2, 3, 9, 22]. However, discrepancies remain between the experimental results from animal models and the clinical trials on humans.

In both, rat and pig models of hemorrhagic shock, injecting estrogen sulfate after significant blood loss has been shown to improve outcomes as well as having protective effects on both myocardial function and vascular responsiveness. This may in part be due to estrogen increasing the expression of heat shock proteins, which protect cells, and thus improve cardiac function [23–31]. This could serve as a plausible explanation for the reduced incidence of myocardial infarction and cardiac arrest observed in females compared to males within the current study. Furthermore, studies have shown that males experience a higher incidence of sepsis, multiple organ failure, and mortality following traumatic injury due to alterations in the immune response [32–34]. Large-scale analyses have reported decreased survival rates and a higher frequency of infections and sepsis in males following trauma [35, 36]. A registry study involving over 680,000 patients revealed an association between female sex and a decrease in complication and mortality rates following trauma [35]. Another study of more than 30,000 patients found that males were more likely to develop pneumonia following traumatic injury [36]. These findings align with the results of the current study where the risk of post traumatic ARDS and pneumonia are higher in males.

A number of studies have been conducted to examine if there are sex-related disparities in mortality rates after a spinal cord injury [11, 12, 15–17]. However, these studies have produced varied outcomes. For instance, Furlan et al. recently published a retrospective cohort study on patients with traumatic spinal cord injuries, composed of 5,571 individuals, and could not demonstrate any sex-dependent difference in regard to in-hospital mortality [11]. Another study published in 2019, including 504 individuals, also concluded that there was no significant difference in survival rates between males and females following acute spinal injury [12]. Furthermore, a Swiss observational cohort study composed of 2,421 individuals with traumatic SCI by Chamberlain et al., observed that males had a greater mortality rate than females with a univariate analysis hazard ratio of 1.38 (95% CI 1.10–1.74). However, the multivariable analysis resulted in a hazard ratio of 1.0 (95% CI 0.79–1.10), which was not statistically significant [15]. On the other hand, Hatch et al. documented a greater mortality rate among males when performing an analysis with a multivariable Cox proportional hazards model (HR 1.3 [95% CI 1.0, 1.6]) in a cohort study composed of 535 cases with nontraumatic SCI and 221 cases with traumatic SCI [16]. A larger retrospective cohort study from Japan also demonstrated a significantly higher mortality risk among males (odds ratio: 2.06 [95% CI 1.44–2.93]) after multivariable logistic regression analysis using 8069 cases of traumatic SCI [17].

The previously conducted studies on sex-dependent differences after spinal injury have mainly focused on in-hospital mortality and functional outcomes after surgery for traumatic SCI [11, 12, 15–17]. Nevertheless, the current study could also demonstrate a lower risk of complications postoperatively in females after adjusting for SCI in isolated TSI. The differences seen in the mentioned studies might be due to the complexity in the relationship between biological sex and adverse outcomes, which may also be influenced by various factors, such as methodological variances, differences in healthcare systems, and population profiles.

Our study has several advantages, including the use of a comparatively large national sample population composed of patients treated at over 875 trauma centers across the United States. In contrast to previous studies, the current investigation has included all TSIs undergoing surgery. However, due to the retrospective nature of this study, it should be acknowledged that the study's findings are only observed associations and further research is required to further investigate possible causal relationships and mechanisms. Other limitations to acknowledge are the risk of potential residual confounding, dependency on the precision of data recorded in the dataset, as well as the inability to evaluate variables not present in the dataset, such as results of blood samples taken on admission, the cause of death, the utilization and extent of preoperative optimization, and intraoperative factors. Despite these limitations, the large dataset enabled adjustments to be made for a significant number of preadmission comorbidities, racial and demographic differences.

## Conclusion

Female sex is associated with a significantly decreased risk of in-hospital mortality as well as complications following surgical management of traumatic spinal injury. Additional research is required to better understand the underlying mechanism behind this relationship.

### Supplementary Information

Below is the link to the electronic supplementary material.

Supplementary file1 (DOCX 26 kb)

## Data Availability

All data is available for retrieval upon reasonable request.

## References

[1] Kumar R, Lim J, Mekary RA, Rattani A, Dewan MC, Sharif SY (2018). Traumatic spinal injury: global epidemiology and worldwide volume. World Neurosurg.

[2] Choudhry MA, Bland KI, Chaudry IH (2007). Trauma and immune response–effect of gender differences. Injury.

[3] Sperry JL, Nathens AB, Frankel HL, Vanek SL, Moore EE, Maier RV (2008). Characterization of the gender dimorphism after injury and hemorrhagic shock: are hormonal differences responsible?. Crit Care Med.

[4] Angele MK, Frantz MC, Chaudry IH (2006). Gender and sex hormones influence the response to trauma and sepsis: potential therapeutic approaches. Clin Sao Paulo Braz.

[5] Knöferl MW, Angele MK, Diodato MD, Schwacha MG, Ayala A, Cioffi WG (2002). Female sex hormones regulate macrophage function after trauma-hemorrhage and prevent increased death rate from subsequent sepsis. Ann Surg.

[6] Magnotti LJ, Fischer PE, Zarzaur BL, Fabian TC, Croce MA (2008). Impact of gender on outcomes after blunt injury: a definitive analysis of more than 36,000 trauma patients. J Am Coll Surg.

[7] Trentzsch H, Nienaber U, Behnke M, Lefering R, Piltz S (2014). Female sex protects from organ failure and sepsis after major trauma haemorrhage. Injury.

[8] Yang KC, Zhou MJ, Sperry JL, Rong L, Zhu XG, Geng L (2014). Significant sex-based outcome differences in severely injured Chinese trauma patients. Shock Augusta Ga..

[9] Frink M, Pape HC, van Griensven M, Krettek C, Chaudry IH, Hildebrand F (2007). Influence of sex and age on mods and cytokines after multiple injuries. Shock Augusta Ga.

[10] Pape M, Giannakópoulos GF, Zuidema WP, de Lange-Klerk ESM, Toor EJ, Edwards MJR (2019). Is there an association between female gender and outcome in severe trauma? A multi-center analysis in the Netherlands. Scand J Trauma Resusc Emerg Med..

[11] Furlan JC, Shen T, Kurban D (2023). Sex-related discrepancies in the access to optimal care and outcomes after traumatic spinal cord injury: a retrospective cohort study using data from a Canadian Registry. Arch Phys Med Rehabil.

[12] Furlan JC, Craven BC, Fehlings MG (2019). Sex-related discrepancies in the epidemiology, injury characteristics and outcomes after acute spine trauma: a retrospective cohort study. J Spinal Cord Med.

[13] Furlan JC, Krassioukov AV, Fehlings MG (2005). The effects of gender on clinical and neurological outcomes after acute cervical spinal cord injury. J Neurotrauma.

[14] Noe BB, Stapelfeldt CM, Parner ET, Mikkelsen EM (2017). Survival after traumatic spinal cord injury in Denmark: a hospital-based study among patients injured in 1990–2012. Spinal Cord.

[15] Chamberlain JD, Deriaz O, Hund-Georgiadis M, Meier S, Scheel-Sailer A, Schubert M (2015). Epidemiology and contemporary risk profile of traumatic spinal cord injury in Switzerland. Inj Epidemiol.

[16] Hatch BB, Wood-Wentz CM, Therneau TM, Walker MG, Payne JM, Reeves RK (2017). Factors predictive of survival and estimated years of life lost in the decade following nontraumatic and traumatic spinal cord injury. Spinal Cord.

[17] Shibahashi K, Nishida M, Okura Y, Hamabe Y (2019). Epidemiological state, predictors of early mortality, and predictive models for traumatic spinal cord injury: a multicenter nationwide cohort study. Spine.

[18] Wang Y, Guo Z, Fan D, Lu H, Xie D, Zhang D (2018). A meta-analysis of the influencing factors for tracheostomy after cervical spinal cord injury. BioMed Res Int.

[19] WMA - The World Medical Association-WMA Declaration of Helsinki—ethical principles for medical research involving human subjects [Internet]. [citerad 21 september 2020]. Available from: https://www.wma.net/policies-post/wma-declaration-of-helsinki-ethical-principles-for-medical-research-involving-human-subjects/. Accessed 30 Nov 2022.

[20] Morris JA, Gardner MJ (1988). Calculating confidence intervals for relative risks (odds ratios) and standardised ratios and rates. Br Med J Clin Res Ed..

[21] R Development Core Team. R: a language and environment for statistical computing [Internet]. Vienna, Austria: R Foundation for Statistical Computing; 2008. Available from: http://www.R-project.org/. Accessed 30 Nov 2022.

[22] Bösch F, Angele MK, Chaudry IH (2018). Gender differences in trauma, shock and sepsis. Mil Med Res.

[23] Hubbard W, Keith J, Berman J, Miller M, Scott C, Peck C (2015). 17α-Ethynylestradiol-3-sulfate treatment of severe blood loss in rats. J Surg Res.

[24] Miller M, Keith J, Berman J, Burlington DB, Grudzinskas C, Hubbard W (2014). Efficacy of 17α-ethynylestradiol-3-sulfate for severe hemorrhage in minipigs in the absence of fluid resuscitation. J Trauma Acute Care Surg.

[25] Li T, Xiao X, Zhang J, Zhu Y, Hu Y, Zang J (2014). Age and sex differences in vascular responsiveness in healthy and trauma patients: contribution of estrogen receptor-mediated Rho kinase and PKC pathways. Am J Physiol Heart Circ Physiol.

[26] Soliman M (2015). Protective effects of estradiol on myocardial contractile function following hemorrhagic shock and resuscitation in rats. Chin Med J (Engl).

[27] Dhamad AE, Zhou Z, Zhou J, Du Y (2016). Systematic proteomic identification of the heat shock proteins (Hsp) that interact with estrogen receptor alpha (ERα) and biochemical characterization of the ERα-Hsp70 interaction. PLoS ONE.

[28] Wang JL, Ke DS, Lin MT (2005). Heat shock pretreatment may protect against heatstroke-induced circulatory shock and cerebral ischemia by reducing oxidative stress and energy depletion. Shock Augusta Ga.

[29] Hsu JT, Hsieh YC, Kan WH, Chen JG, Choudhry MA, Schwacha MG (2007). Role of p38 mitogen-activated protein kinase pathway in estrogen-mediated cardioprotection following trauma-hemorrhage. Am J Physiol Heart Circ Physiol.

[30] Szalay L, Shimizu T, Suzuki T, Yu HP, Choudhry MA, Schwacha MG (2006). Estradiol improves cardiac and hepatic function after trauma-hemorrhage: role of enhanced heat shock protein expression. Am J Physiol Regul Integr Comp Physiol.

[31] Yu HP, Shimizu T, Choudhry MA, Hsieh YC, Suzuki T, Bland KI (2006). Mechanism of cardioprotection following trauma-hemorrhagic shock by a selective estrogen receptor-beta agonist: up-regulation of cardiac heat shock factor-1 and heat shock proteins. J Mol Cell Cardiol.

[32] Baue AE (2006). MOF, MODS, and SIRS: what is in a name or an acronym?. Shock Augusta Ga.

[33] Shukla A, Hashiguchi N, Chen Y, Coimbra R, Hoyt DB, Junger WG (2004). Osmotic regulation of cell function and possible clinical applications. Shock Augusta Ga.

[34] Ulloa L, Tracey KJ (2005). The ”cytokine profile”: a code for sepsis. Trends Mol Med.

[35] Haider AH, Crompton JG, Oyetunji T, Stevens KA, Efron DT, Kieninger AN (2009). Females have fewer complications and lower mortality following trauma than similarly injured males: a risk adjusted analysis of adults in the National Trauma Data Bank. Surgery.

[36] Gannon CJ, Pasquale M, Tracy JK, McCarter RJ, Napolitano LM (2004). Male gender is associated with increased risk for postinjury pneumonia. Shock Augusta Ga.

