# Supplementary material for: Sex disparities in adverse outcomes after surgically managed isolated traumatic spinal injury
Source: Eur J Trauma Emerg Surg. 2023 May 16;50(1):149–55. doi: 10.1007/s00068-023-02275-z (PMC10923959; doi:10.1007/s00068-023-02275-z)
Supplement: Supplementary file 1 — Supplementary file1 (DOCX 26 kb) [file 68_2023_2275_MOESM1_ESM.docx]

| **Supplemental Table 1.** Demographics, clinical characteristics, and outcomes in patients with isolated traumatic spine injuries, after inverse probability weighting | | | | | | |
| --- | --- | --- | --- | --- | --- | --- |
|  | **Male (N =** **43,741)** | | **Female (N =** **43,930)** | | **ASD** | |
| Age, median [IQR] | 56 [39.0-69.0] | | 56 [37.0-71.0] | | 0.013 | |
| Race, n (%) |  | |  | |  | |
| White | 34,268 (78.3) | | 34,401 (78.3) | | 0.001 | |
| Black | 5,122 (11.7) | | 5,095 (11.6) | | 0.004 | |
| Asian | 959 (2.2) | | 954 (2.2) | | 0.001 | |
| American indian | 369 (0.8) | | 380 (0.9) | | 0.002 | |
| Pacific islander | 124 (0.3) | | 120 (0.3) | | 0.002 | |
| Other | 2,637 (6.0) | | 2,717 (6.2) | | 0.007 | |
| Hypertension, n (%) | 16,742 (38.3) | | 16,642 (37.9) | | 0.008 | |
| Previous myocardial infarction, n (%) | 410 (0.9) | | 443 (1.0) | | 0.007 | |
| Congestive heart failure, n (%) | 1,537 (3.5) | | 1,564 (3.6) | | 0.002 | |
| History of peripheral vascular disease, n (%) | 303 (0.7) | | 317 (0.7) | | 0.003 | |
| Cerebrovascular disease, n (%) | 900 (2.1) | | 894 (2.0) | | 0.002 | |
| Dementia, n (%) | 1,125 (2.6) | | 1,127 (2.6) | | <0.001 | |
| COPD, n (%) | 2,887 (6.6) | | 2,967 (6.8) | | 0.006 | |
| Current smoker, n (%) | 10,339 (23.6) | | 10,420 (23.7) | | 0.002 | |
| Chronic renal failure, n (%) | 591 (1.4) | | 620 (1.4) | | 0.005 | |
| Diabetes mellitus, n (%) | 7,566 (17.3) | | 7,645 (17.4) | | 0.003 | |
| Cirrhosis, n (%) | 383 (0.9) | | 415 (0.9) | | 0.007 | |
| Coagulopathy, n (%) | 1,469 (3.4) | | 1,500 (3.4) | | 0.003 | |
| Currently receiving chemotherapy  for cancer, n (%) | 126 (0.3) | | 128 (0.3) | | 0.001 | |
| Metastatic cancer, n (%) | 254 (0.6) | | 243 (0.6) | | 0.004 | |
| Drug use disorder, n (%) | 2,803 (6.4) | | 2,959 (6.7) | | 0.013 | |
| Alcohol use disorder, n (%) | 3,744 (8.6) | | 3,899 (8.9) | | 0.011 | |
| Major psychiatric illness, n (%) | 4,728 (10.8) | | 4,747 (10.8) | | <0.001 | |
| Advanced directive limiting care, n (%) | 995 (2.3) | | 1,009 (2.3) | | 0.002 | |
| ISS, median [IQR] | | 9 [5.0-16.0] | | 9 [5.0-16.0] | | 0.011 |
| Head AIS, n (%) |  | |  | | 0.004 | |
| Injury not present | 37,623 (86.0) | | 37,723 (85.9) | |  | |
| 1 | 6,118 (14.0) | | 6,206 (14.1) | |  | |
| Face AIS, n (%) |  | |  | | 0.003 | |
| Injury not present | 36,081 (82.5) | | 36,190 (82.4) | |  | |
| 1 | 7,660 (17.5) | | 7,739 (17.6) | |  | |
| Neck AIS, n (%) |  | |  | | <0.001 | |
| Injury not present | 43,157 (98.7) | | 43,342 (98.7) | |  | |
| 1 | 584 (1.3) | | 588 (1.3) | |  | |
| Spine AIS, n (%) |  | |  | | 0.012 | |
| 2 | 16,308 (37.2) | | 16,377 (37.2) | |  | |
| 3 | 16,225 (37.1) | | 16,172 (36.8) | |  | |
| 4 | 8,220 (18.8) | | 8,247 (18.8) | |  | |
| 5 | 2,988 (6.8) | | 3,133 (7.1) | |  | |
| Thorax AIS, n (%) |  | |  | | 0.002 | |
| Injury not present | 41,094 (93.9) | | 41,252 (93.9) | |  | |
| 1 | 2,647 (6.1) | | 2,678 (6.1) | |  | |
| Abdomen AIS, n (%) |  | |  | | 0.005 | |
| Injury not present | 42,511 (97.2) | | 42,659 (97.1) | |  | |
| 1 | 1,230 (2.8) | | 1,270 (2.9) | |  | |
| Upper extremity AIS, n (%) |  | |  | | 0.006 | |
| Injury not present | 39,083 (89.4) | | 39,163 (89.2) | |  | |
| 1 | 4,658 (10.6) | | 4,766 (10.8) | |  | |
| Lower extremity AIS, n (%) |  | |  | | 0.006 | |
| Injury not present | 39,534 (90.4) | | 39,624 (90.2) | |  | |
| 1 | 4,207 (9.6) | | 4,306 (9.8) | |  | |
| External/Other AIS, n (%) |  | |  | | 0.002 | |
| Injury not present | 41,884 (95.8) | | 42,044 (95.7) | |  | |
| 1 | 1,857 (4.2) | | 1,886 (4.3) | |  | |
| Level of spine injury, n (%) |  | |  | |  | |
| Cervical | 26,798 (61.3) | | 26,946 (61.3) | | 0.002 | |
| Thoracic | 12,059 (27.6) | | 12,151 (27.7) | | 0.002 | |
| Lumbar | 11,914 (27.2) | | 12,014 (27.3) | | 0.002 | |
| Spinal cord injury, n (%) |  | |  | |  | |
| Cervical | 13,837 (31.6) | | 14,005 (31.9) | | 0.005 | |
| Thoracic | 2,449 (5.6) | | 2,502 (5.7) | | 0.004 | |
| Lumbar | 2,005 (4.6) | | 2,035 (4.6) | | 0.002 | |
| Level of spine surgery, n (%) |  | |  | |  | |
| Cervical | 31,869 (72.9) | | 31,984 (72.8) | | 0.001 | |
| Thoracic | 23,856 (54.5) | | 23,955 (54.5) | | <0.001 | |
| Lumbar | 19,941 (45.6) | | 20,063 (45.7) | | 0.002 | |
| In-hospital mortality, n (%) | 1,107 (2.5) | | 700 (1.6) | | 0.066 | |
| Myocardial infarction, n (%) | 125 (0.3) | | 91 (0.2) | | 0.016 | |
| Cardiac arrest with CPR, n (%) | 550 (1.3) | | 347 (0.8) | | 0.047 | |
| Stroke, n (%) | 105 (0.2) | | 101 (0.2) | | 0.002 | |
| Deep vein thrombosis, n (%) | 761 (1.7) | | 505 (1.1) | | 0.050 | |
| Pulmonary embolism, n (%) | 375 (0.9) | | 206 (0.5) | | 0.048 | |
| Acute respiratory distress syndrome, n (%) | 328 (0.7) | | 211 (0.5) | | 0.034 | |
| Pneumonia, n (%) | 1,119 (2.6) | | 739 (1.7) | | 0.061 | |
| Surgical site infection, n (%) | 170 (0.4) | | 133 (0.3) | | 0.014 | |
| Duration of ICU stay, median [IQR] | 4 [3.0-7.0] | | 4 [3.0-7.0] | | 0.063 | |
| An ASD <0.1 is considered balanced. Duration of ICU stay is measured in days.  *ASD, absolute standardized difference; COPD, chronic obstructive pulmonary disease; ISS, Injury severity score; AIS, Abbreviated injury severity score; ICU, Intensive care unit* | | | | | | |
